# Different scaling of white matter volume, cortical connectivity, and gyrification across rodent and primate brains

**DOI:** 10.3389/fnana.2013.00003

**Published:** 2013-04-09

**Authors:** Lissa Ventura-Antunes, Bruno Mota, Suzana Herculano-Houzel

**Affiliations:** ^1^Instituto de Ciências Biomédicas, Universidade Federal do Rio de JaneiroIlha do Fundão, Brazil; ^2^Instituto Nacional de Neurociência Translacional, Ministério de Ciência de Tecnologia/CNPqRio de Janeiro, Brazil

**Keywords:** white matter, number of neurons, allometry, brain size, cortical expansion, gyrification

## Abstract

Expansion of the cortical gray matter in evolution has been accompanied by an even faster expansion of the subcortical white matter volume and by folding of the gray matter surface, events traditionally considered to occur homogeneously across mammalian species. Here we investigate how white matter expansion and cortical folding scale across species of rodents and primates as the gray matter gains neurons. We find very different scaling rules of white matter expansion across the two orders, favoring volume conservation and smaller propagation times in primates. For a similar number of cortical neurons, primates have a smaller connectivity fraction and less white matter volume than rodents; moreover, as the cortex gains neurons, there is a much faster increase in white matter volume and in its ratio to gray matter volume in rodents than in primates. Order-specific scaling of the white matter can be attributed to different scaling of average fiber caliber and neuronal connectivity in rodents and primates. Finally, cortical folding increases as different functions of the number of cortical neurons in rodents and primates, scaling faster in the latter than in the former. While the neuronal rules that govern gray and white matter scaling are different across rodents and primates, we find that they can be explained by the same unifying model, with order-specific exponents. The different scaling of the white matter has implications for the scaling of propagation time and computational capacity in evolution, and calls for a reappraisal of developmental models of cortical expansion in evolution.

## Introduction

Across different mammalian orders and species, adult brain cerebral cortices vary over several orders of magnitude in size, becoming more folded as their size increases, and gaining proportionally more white than gray matter (Hofman, 1985; Welker, 1990; Zhang and Sejnowski, 2000). Cortical folding has been considered to increase with brain size due to gray matter lateral expansion constrained by the interior surface of the skull (Welker, 1990), resulting in the underlying white matter being pushed inwards (Le Gros Clark, 1945), and a faster increase in the number of neurons than would occur if the cortex expanded isometrically. Because the distribution of neurons beneath the cortical surface has traditionally been considered to be constant across species ever since the work of Rockel et al. (1980), cortical folding would thus be a direct function of the number of neurons in the cortex. This is consistent with the usual expectation that elephants and large cetaceans, with larger brains than humans and the most gyrencephalic mammalian brains (Manger et al., 2012), also have larger numbers of cortical neurons. Along the same lines, white matter volume has been considered to scale identically as a function of gray matter volume across different mammalian species, assuming that neuronal density scales similarly with brain size, and that the percentage of cortical neurons connected through the white matter (i.e., cortical connectivity) remains constant across species (Prothero, 1997; Zhang and Sejnowski, 2000; Wang et al., 2008).

Contrary to these traditional views, however, we have recently shown that mammalian cortical size is not a uniform function of the number of cortical neurons (reviewed in Herculano-Houzel, 2011), and neither is cortical connectivity constant across primates (Herculano-Houzel et al., 2010). Using the isotropic fractionator to count neurons (Herculano-Houzel and Lent, 2005), we find that cortical mass increases much faster as a function of neuronal numbers in rodents than in primates (Herculano-Houzel et al., 2006, 2007, 2011; Gabi et al., 2010), as average neuronal cell mass increases quickly as a function of neuronal numbers in rodents, but hardly changes in primates. Thus, neuronal densities are larger in primate cortices than in rodent cortices of a comparable size (Herculano-Houzel, 2011). Moreover, the number of neurons beneath the cortical surface is not constant across primate species as previously thought (Herculano-Houzel et al., 2008). The combination of these findings leaves no reason to expect cortical folding to be a simple universal function of cortical neuronal numbers for all mammals. For instance, although large cetaceans and artiodactyls have more folded cerebral cortices primates with similarly sized brains (Pillay and Manger, 2007), they probably have not more but rather far fewer cortical neurons than primates, due to much lower neuronal densities (Tower, 1954; Herculano-Houzel, 2009; and our unpublished observations). Moreover, given the different relationships between gray matter volume and number of neurons across mammalian orders, it remains to be determined whether white matter scaling is indeed shared across mammals as it is assumed to be (Zhang and Sejnowski, 2000). The scaling of cortical connectivity through the white matter as a function of the number of cortical neurons is a basic issue in functional neuroanatomy, with important functional, developmental, and evolutionary consequences that, to the extent of our knowledge, has often been modeled, but never examined directly.

Here we examine whether the scaling relationship between white and gray matter is indeed universal across mammals by comparing how white matter properties, such as volume, fraction of cortical neurons connected, and average axonal caliber scale as a function of the number of neurons in the gray matter of primate and rodent species. We also test our hypothesis that cortical folding is driven by a shared, conserved mechanism across mammalian species, not as a simple function of the number of cortical neurons, but of the number of cortical neurons with axons through the white matter and of the average caliber and longitudinal tension along these axons (Mota and Herculano-Houzel, 2012).

## Materials and methods

The goal of this study required determining total numbers of neuronal and non-neuronal cells in the gray and white matter of rodent cerebral cortices, in order to examine their scaling rules in a way that would be comparable with primates. In principle, we should have had to determine these numbers in a new set of animals, given that our initial quantification of cell numbers in rodents applied to the combined gray and white matter of the cerebral cortex (Herculano-Houzel et al., 2006). Instead, we used the unprocessed cerebral hemispheres left from our initial study to examine the same animals. Because these had been fixed for too long, however, determining numbers of neurons directly by immunocytochemistry was no longer possible. Therefore, we resorted to carefully dissecting the white and gray matter from these remaining cortical hemispheres; determining the total numbers of cells in the white matter; and subtracting these numbers from the average of the original data for the combined gray and white matter. We consider that the vast majority of cells in the white matter are non-neuronal, and therefore the total numbers of cells found in the white matter in the present study are referred to as “other cells” (*O*_*W*_); and, by subtracting the average number of cortical neurons found in the original study from the total numbers of cells found in the gray matter in the present study, we consider that the remaining number corresponds to the total of other, non-neuronal cells in the cortical gray matter (*O*_*G*_).

### Experimental animals

We analyzed five of the six species examined originally (Herculano-Houzel et al., 2006): mouse (two hemispheres, Ca50 and Ca54), rat (two hemispheres, RaL01), guinea pig (two hemispheres, Co02 and Co03), agouti (one hemisphere, CuF), and capybara (one hemisphere, Hy02). Only the rat was not in the original dataset; for the other four species, we analyzed the remaining hemisphere of the individuals examined originally.

### Comparison to primate dataset

Here we use the published dataset of ten species of primates plus tree shrew as in Herculano-Houzel et al. (2008, 2010) for which we determined the cellular composition of the white and gray matter separately. The tree shrew (*Tupaia belangeri*), although a scandentian and not a primate, is included in the “primate” dataset for the sake of consistency with prior analyses. This is warranted because the inclusion or exclusion of the tree shrew to the primate dataset does not affect the results (Herculano-Houzel et al., 2008, 2010). Besides the tree shrew, the species included are galago (*Otolemur garnetti*), marmoset (*Callithrix jacchus*), owl monkey (*Aotus trivirgatus*), squirrel monkey (*Saimiri sciureus*), capuchin monkey (*Cebus apella*), baboon (*Papio* sp.), Goeldi's marmoset (*Callimico goeldii*), long-tailed macaque monkey (*Macaca fascicularis*), and bonnet macaque monkey (*Macaca radiata*). This dataset does not include human brains (Azevedo et al., 2009), for which *V*_*W*_, *A*_*W*_, and *O*_*W*_ were not available. For this reason, some of the exponents found here do not match exactly the exponents reported in recent reviews for the full dataset (Herculano-Houzel, 2011, 2012).

### Disection

All hemispheres had been fixed for over 3 years in 4% paraformaldehyde in phosphate buffer 0.1 M. Each fixed cortical hemisphere was embedded in 3% agar and sliced manually in a coronal series of 1 mm (mouse) or 2 mm sections (other species). Each section was digitalized at 1200 dpi on a flatbed scanner for surface area reconstruction. Cortical white and gray matter, including the hippocampus, were then dissected apart in each section under a dissecting scope, weighed, and the white matter was processed separately with the isotropic fractionator (Herculano-Houzel and Lent, 2005) to determine total cell count numbers for each section.

### Surface and volume reconstruction

The perimeter of the pial surface of the cerebral cortex and the gray matter area in the coronal plane of each section were traced and measured from the scanned images using Canvas X software (ACD Systems). The same procedure was applied to the calculation of the perimeter of the interface between white and gray matter (i.e., the white matter surface) and of the area of subcortical white matter in the coronal plane (between cortical gray matter and striatum) in each section.

We next used these values of perimeter and coronal area for each section to reconstruct the total volume and surface area of the gray and white matter. Since we did not have the complete 3D coordinates of each point in both tracings (which would allow a more precise reconstruction by triangulation), we simply assume that adjacent slices have approximately congruent shapes, differing only by some uniform scaling factor. Using this approximation, it can be shown that, for the *i*-th section contained between slices *i* and *i* + 1, the volume and lateral surface area are given by

$$\begin{array}{l} A={\left\{{\left(A_{i}-A_{i+1}\right)}^{2}+{\left[h\left(P_{i}+P_{i+1}\right)/2\right]}^{2}\right\}}^{1/2}\\ V=h\left[A_{i}+A_{i\;+1}+{\left(A_{i}A_{i+1}\right)}^{1/2}\right]/3 \end{array}$$

where *A* is total surface area and *V* is the total volume for the reconstructed white or gray matter, *A*_*i*_ and *A*_*i* + 1_ (or *P*_*i*_ and *P*_*i* + 1_) are the coronal section surface areas (or perimeters) for two adjacent sections, and h is the section thickness. We assume that the last section converges on a cusp (thus implicitly assuming that the (*n* + 1)-th section is a point with null perimeter and area). This method provides a good approximation of surface area and volume across sections even if there is a systematic variation in the shape of the sections, as long as this variation is small between one section and the next.

Average cortical thickness was then estimated as the ratio between the total gray matter volume and surface area in each specimen. The gray matter folding index was calculated by first tracing the exposed surface of the cortex (the smallest surface of gray matter that did not enter sulci), then estimating the total area of the exposed cortical surface as above, and dividing the total gray matter surface area by that value.

All values reported (Table 1) refer to a single hemisphere, as in Herculano-Houzel et al. (2008, 2010).

**Table 1:** **Average volume and cellular composition of the cortical gray and white matter**.

**Species**	***V*_*G*_**	***V*_*W*_**	***A*_*G*_**	***A*_*W*_**	***T*_*G*_**	***N*_*G* + *W*_**	***O*_*W*_**	***F*_*E*_**
*Mus musculus*	107.11 ± 44.40	8.78 ± 5.25	147.77 ± 35.91	81.80 ± 40.74	0.749 ± 0.115	6.845 × 10^6^	3.696 × 10^6^	1.02
*Rattus norvegicus*	430.82 ± 41.44	64.60 ± 8.27	355.54 ± 26.55	271.72 ± 70.58	1.248 ± 0.008	15.510 × 10^6^	12.928 × 10^6^	1.04
*Cavia porcellus*	812.43 ± 109.97	94.47 ± 20.30	571.18 ± 49.58	341.40 ± 42.24	1.584 ± 0.058	21.755 × 10^6^	27.318 × 10^6^	1.04
*Dasyprocta primnolopha*	2607.84	672.52	1412.67	1257.80	1.911	56.320 × 10^6^	118.097 × 10^6^	1.17
*Hydrochoerus hydrochoeris*	16896.51	4987.28	6004.54	4986.58	3.089	153.150 × 10^6^	707.518 × 10^6^	1.30
*Tupaia glis*	531.9	181.8	497.0	323.8	1.09	21.95 ± 1.60 × 10^6^	16.22 × 10^6^	1.04
*Callithrix jacchus*	2036.1	887.1	1534.0	1087.6	1.31	120.33 ± 43.30 × 10^6^	74.63 × 10^6^	1.18
*Otolemur garnetii*	2492.0	1060.3	1745.0	1235.4	1.46	88.50 ± 14.75 × 10^6^	102.89 × 10^6^	1.25
*Aotus trivirgatus*	3295.4	1265.2	2214.0	1588.0	1.50	200.32 ± 67.34	170.04 × 10^6^	1.36
*Callimico goeldii*	3125.1	1413.4	1953.0	1470.1	1.60	178.77 × 10^6^	157.13 × 10^6^	1.26
*Saimiri sciureus*	7706.7	4452.5	5250.0	4377.8	1.47	645.73 ± 43.74 × 10^6^	353.05 × 10^6^	1.57
*M. fascicularis*	13234.4	11911.2	9381.0	7920.1	1.41	400.74 × 10^6^	484.40 × 10^6^	1.65
*M. radiata*	13220.4	9114.1	8441.0	7289.9	1.57	829.60 × 10^6^	841.38 × 10^6^	1.81
*Cebus apella*	13475.6	8220.5	7653.0	6025.0	1.77	930.67 ± 507.78 × 10^6^	868.73 × 10^6^	1.69
*Papio cynocephalus*	33910.3	17814.2	16689.0	13963.4	2.03	1420.34 ± 18.07 × 10^6^	1860.00 × 10^6^	1.92

All values are average ± standard deviation and refer to one cerebral cortical hemisphere only. V_G_, volume of the cortical gray matter (mm^3^); V_W_, volume of the subcortical white matter (mm^3^); A_G_, surface area of the cortical gray matter (mm^2^); A_W_, surface area of the subcortical white matter (i.e., of the gray-white matter interface, in mm^2^); T_G_, thickness of the gray matter (mm); N_G + W_, total number of neurons in the cerebral cortex (GM and occasional neurons found in the WM; O_W_, number of other (non-neuronal) cells in the WM; F_G_, folding index of the GM (total GM surface/exposed GM surface).

## Results

**Figure 1:**
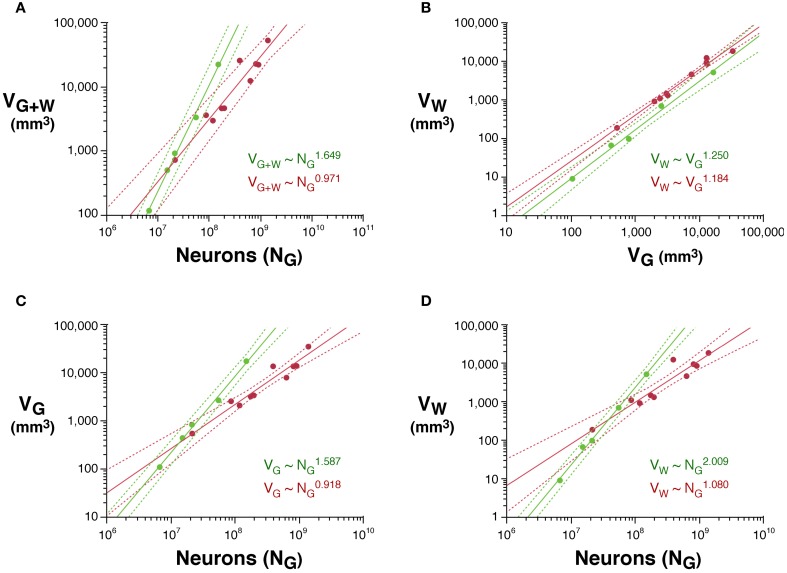
**Scaling of gray and white matter volumes (*V*_*G*_ and *V*_*W*_, respectively) and total cortical volume (*V*_*G* + *W*_) in rodents (green) and primates (red). (A)** Total cortical volume increases more steeply with increasing numbers of cortical neurons in rodents than in primates. **(B)** White matter volume increases as non-overlapping functions of gray matter volume in rodents and primates. **(C)** Gray matter volume increases faster with increasing numbers of cortical neurons in rodents than in primates. **(D)** White matter volume also increases faster with increasing numbers of cortical neurons in rodents than in primates. Each point represents one species. Power functions are plotted for each mammalian order with the respective 95% confidence intervals (dotted lines). Exponents are indicated.

The distributions of volumes of gray and white matter in the cerebral cortex are largely overlapping across rodent and primate species in our dataset (Figure 1B), which allows for direct comparison of the scaling of their cellular composition. The total volume of the cerebral cortex, *V*_*G* + *W*_, expressed as the sum of the volumes of the gray and white matter, scales with the number of cortical neurons *N*_*G*_, as *V*_*G* + *W*_ ~ *N*^*v*^_*G*_, much faster in rodents than in primates. In the sampled rodents, we find a *v* of 1.649 (95% CI, 1.539–1.759, *p* < 0.0001), while in primates *v* is 0.971 (95% CI, 0.779–1.163, *p* < 0.0001; Figure 1A). Both values match well the exponents of 1.699 and 1.087, respectively, obtained previously for a larger number of species (Herculano-Houzel et al., 2006, 2007, 2011; Gabi et al., 2010; reviewed in Herculano-Houzel, 2011).

### Gray matter

The volume of the gray matter alone also increases more rapidly as a function of *N*_*G*_ in rodents (*V*_*G*_ ~ *N*^1.587^_*G*_; 95% CI, 1.459–1.715, *p* = 0.0001, *r*^2^ = 0.993; and therefore, D_N_ ~ N^−0.587^) than in primates (*V*_*G*_ ~ *N*^0.918^_*G*_, 95% CI, 0.752–1.084, *p* < 0.0001, *r*^2^ = 0.990). As a result, for the measured range of cerebral masses, primate cortices contain more neurons than rodent cortices with similar gray matter volume, and the discrepancy increases with volume (Figure 1C).

**Figure 2:**
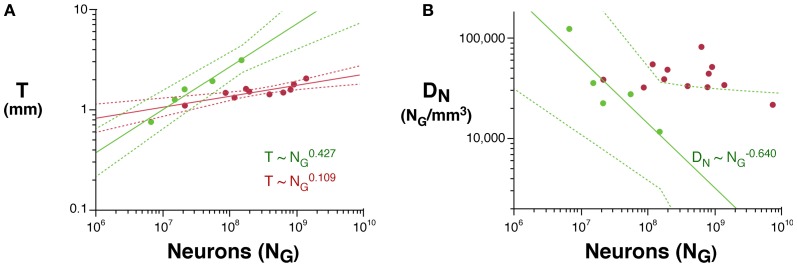
**Scaling of average cortical thickness (A) and of average neuronal density (B) as a function of numbers of cortical neurons in rodents (green) and primates (red).** Power functions, where the exponents are significant at the level of *p* < 0.05, are plotted for each mammalian order with the respective 95% confidence intervals (dotted lines). Exponents are indicated.

Cortical thickness increases rapidly with *N*^0.427^_*G*_ across rodent species (95% CI, 0.329–0.525, *p* = 0.0032), in contrast to a slow but still significant increase with *N*^0.109^_*G*_ across the primate species in the dataset (95% CI, 0.059–0.159, *p* = 0.0022). The direct comparison of the two distributions shows that, for the same number of cortical neurons or gray matter volume, the cerebral cortex is thicker in rodents than in primates, and its thickness increases faster in rodent than in primate cortices as both gain neurons (Figure 2A).

The fast increase in cortical thickness across rodents is accompanied by a significant and rapid decrease in neuronal density, with *N*^−0.640^_*G*_ (95% CI, −1.024 to −0.256, *p* = 0.0445), while neuronal density does not vary significantly with *N*_*G*_ across primate cortices (exponent, −0.062, *p* = 0.3807; Figure 2B). This suggests that faster cortical thickening in rodents in comparison to primates is at least partly related to the average size of the neurons increasing in the former, while remaining nearly invariant in the latter.

**Figure 3:**
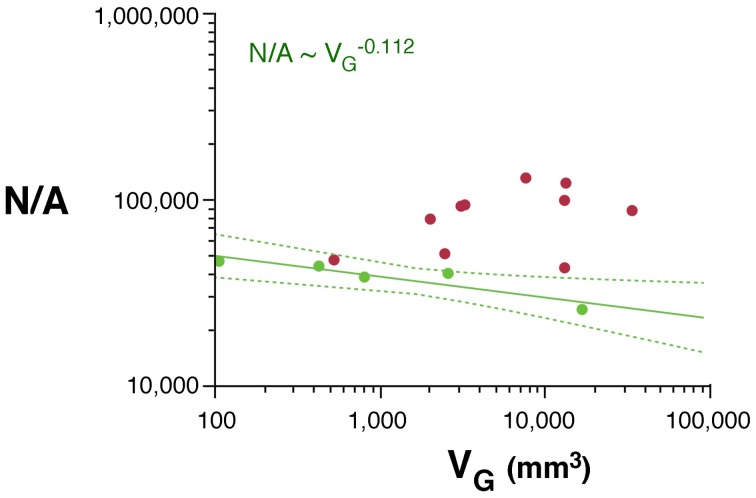
**Scaling of average number of neurons underneath 1 mm^2^ of cortical gray matter as a function of gray matter volume in rodents (green) and primates (red).** The power function for rodents is plotted with the respective 95% confidence interval (dotted lines).

Across rodents, the decrease in neuronal density at a faster rate than the increase in cortical thickness as a function of *N*_*G*_ implies that cortical neurons become spread more thinly along the cortical surface, with a smaller N/A in larger cortices. Indeed, we find that N/A varies 1.8× between mouse and capybara (46,322 and 25,506 neurons/mm^2^, respectively), decreasing with increasing *V*_*G*_ as N/A ~ *V*^−0.112^_*G*_ (95% CI, −0.168 to −0.056, *p* = 0.0291, *r*^2^ = 0.784; Figure 3), and with *N*^−0.174^_*G*_ (95% CI, −0.204 to −0.140, *p* = 0.0414). According to our model of cortical scaling (Mota and Herculano-Houzel, 2012), this is compatible with (1) increasing connectivity and/or (2) increasing axonal caliber as the rodent cortex gains neurons. In primates, in contrast, although average N/A varies 3× across primate species, it does not vary systematically with *N*_*G*_ (Herculano-Houzel et al., 2010; Figure 3).

### White matter

**Figure 4:**
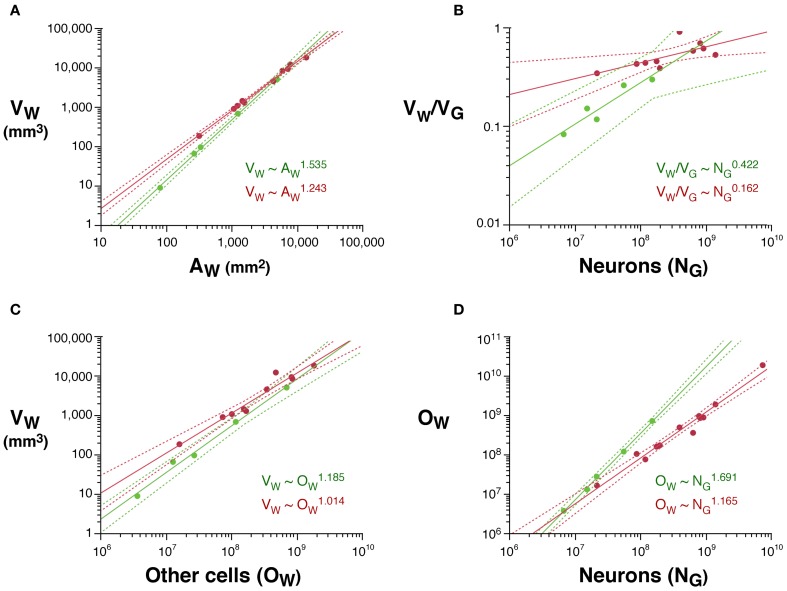
**Scaling laws for the white matter. (A)** White matter volume scales differently with increasing surface area of the white matter (*A*_*W*_) in rodents (green) than in primates (red). **(B)** White/gray matter volume ratio increases faster in rodents than in primates with increasing numbers of cortical neurons. **(C)** White matter volume scales faster with increasing numbers of other cells (*O*_*W*_, presumably mostly oligodendrocytes) in the white matter in rodents than in primates. **(D)** The number of other cells in the white matter increases much faster with increasing numbers of cortical neurons in rodents than in primates. Each point represents one species. Power functions are plotted for each mammalian order with the respective 95% confidence intervals (dotted lines). Exponents are indicated.

As would be expected for the relationship between volume and surface area of an isometrically expanding volume, we find that, in rodents, *V*_*W*_ increases with approximately *A*^3/2^_*W*_ (*V*_*W*_ ~ *A*^1.535^_*W*_; 95% CI, 1.475–1.595, *p* < 0.0001, *r*^2^ = 0.998). In contrast, *V*_*W*_ increases less than expected with *A*_*W*_ in primates (*V*_*W*_ ~ *A*^1.243^_*W*_; 95% CI, 1.181–1.305, *p* < 0.0001, *r*^2^ = 0.994), significantly below isometricity (Figure 4A).

The white matter increases in volume as a function of gray matter volume with fairly similar rates across rodents (*V*_*W*_ ~ *V*^1.250^_*G*_; 95% CI, 1.136–1.384, *p* = 0.0003, *r*^2^ = 0.990) and primates (*V*_*W*_ ~ *V*^1.184^_*G*_; 95% CI, 1.076–1.292; Figure 1B). However, the two relationships are non-overlapping, which becomes more evident when plotted on a linear scale (not shown), such that for a similar *V*_*G*_, *V*_*W*_ is always larger in primates than in rodents (Figure 1B). While the ratio *V*_*W*/_*V*_*G*_ is higher in all primates than in rodents, and also higher in the former for a same number of cortical neurons (Figure 4B), it increases more rapidly with the number of cortical neurons in rodents (with N^0.422^; 95% CI, 0.250–0.594, *p* = 0.0164, *r*^2^ = 0.851) than in primates (with N^0.162^; 95% CI, 0.048–0.276, *p* = 0.0217, *r*^2^ = 0.440; Figure 4B).

The apparently similar scaling of white matter volume with gray matter volume across rodents and primates, therefore, obscures very different scaling relationships between *V*_*W*_ and number of neurons. *V*_*W*_ increases rapidly across rodents with *N*^2.009^_*G*_ (95% CI, 1.879–2.139, *p* < 0.0001, *r*^2^ = 0.996), but it scales across primates with only *N*^1.080^_*G*_ (95% CI, 0.84–1.320, *p* < 0.0001, *r*^2^ = 0.898; Figure 1D). Thus, the addition of neurons to the cerebral cortex is accompanied by a much faster increase in white matter volume in the rodent than in the primate cortex.

Increases in white matter volume can be due to larger total axonal length, to wider average axon caliber, or both. The number of other (non-neuronal) cells in the white matter, *O*_*W*_, can be considered a proxy for the total length of myelinated axons in the white matter, L (Barres and Raff, 1994, 1999). We find that *V*_*W*_ scales as different, non-overlapping functions of *O*_*W*_ across rodents (*V*_*W*_ ~ *O*^1.185^_*W*_; 95% CI, 1.065–1.305, *p* = 0.0003, *r*^2^ = 0.990) and primates (*V*_*W*_ ~ *O*^1.014^_*W*_; 95% CI, 0.854–1.174, *p* < 0.0001, *r*^2^ = 0.946; Figure 4C). Because *V*_*W*_ is the product of L and average axonal caliber in the white matter, *a*, this suggests that *a* increases together with *V*_*W*_ in rodents, but not in primates. *O*_*W*_ also increases much faster with the number of cortical neurons in rodents (*O*_*W*_ ~ *N*^1.691^_*G*_; 95% CI, 1.619–1.763, *p* < 0.0001, *r*^2^ = 0.969) than in primates (*O*_*W*_ ~ *N*^1.165^_*G*_; 95% CI, 1.041–1.289, *p* < 0.0001, *r*^2^ = 0.969; Figure 4D).

### Estimating changes in connectivity and axonal caliber

The scaling relationships that apply to the white matter allow the estimation of how the fraction of *N*_*G*_ connected through the white matter, *n*, and the average fiber caliber in the white matter, *a*, vary with *N*_*G*_, assuming that these are power functions of *N*_*G*_, as we have shown before (Mota and Herculano-Houzel, 2012). Briefly, the total length L of all axons in the white matter, given by L = *n*, *N*, *l*, is considered proportional to the total number of oligodendrocytes found in the white matter (Barres and Raff, 1994, 1999), which we assume to be well approximated by the total number of non-neuronal cells in the white matter, *O*_*W*_. Thus, as mentioned above, *O*_*W*_ ~ *L*. Now, because *V*_*W*_ amounts to L multiplied by a, then *V*_*W*_ ~ *O*_*W*_ *a*. Expressing *O*_*W*_ as an empirically determined power function of *N*_*G*_, *O*_*W*_ ~ *N*^ω^_*G*_, and *a* as proportional to *N*^α^_*G*_, then *V*_*W*_ may be rewritten as *V*_*W*_ ~ *N*^ω^_*G*_ *N*^α^_*G*_.

Given that in rodents we find *V*_*W*_ ~ *N*^2.009^_*G*_ and *O*_*W*_ ~ *N*^1.691^_*G*_, then α = 2.009–1.691 = 0.318 ± 0.074. The average axonal caliber in the white matter thus increases quite rapidly as the rodent cortex gains neurons, scaling with *N*^0.318 ± 0.074^_*G*_. This is significantly different from the exponent of 0.032 ± 0.104, which is not significantly different from zero (obtained using M_W_ instead of *V*_*W*_, which was not available for all species; Herculano-Houzel et al., 2010). Using M_W_ as a proxy for *V*_*W*_ and *N*_*G*_ to calculate α does not lead to significantly different results in rodents (M_W_ ~ *N*^1.991^_*G*_, 95% CI, 1.863–2.119; α = 0.300).

The scaling of the fraction of cortical neurons connected through the white matter (the connectivity fraction, *n*) as a power law of N such that *n* ~ *N*^*c*^_*G*_ can next be estimated from the empirical relationship between *A*_*W*_ and *N*_*G*_, *A*_*W*_ ~ *nN*_*G*_*a*. Across rodents, we find that *A*_*W*_ ~ *N*^1.308^ (95% CI, 1.230–1.384, *p* < 0.0001), which is significantly different from the relationship observed in primates, of *A*_*W*_ ~ *N*^0.873^ (95% CI, 0.695–1.051, *p* < 0.0001). Replacing *n* and *a* by their respective power laws of *N*_*G*_, we get *A*_*W*_ ~ *N*^*c*^_*G*_ *N*_*G*_ *N*^α^_*G*_ ~ *N*^*c* + 1+ α^_*G*_. Since in rodents *A*_*W*_ ~ *N*^1.308^_*G*_ and α = 0.318 ± 0.074, then *c* = −0.010 ± 0.083, which is not significantly different from zero. The rodent cerebral cortex thus gains neurons without changing its fraction of neurons connected through the white matter. This is in contrast to a value of *c* = 0.159 ± 0.137 in primates, which suggests that cortical connectivity through the white matter decreases across species as the primate cortex gains neurons.

**Figure 5:**
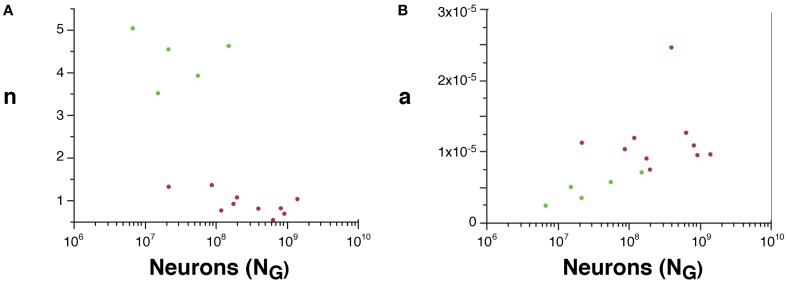
**Scaling of the connectivity fraction, *n***(A)** and of average axonal caliber **(B)** as a function of numbers of cortical neurons in rodents (green) and primates (red). (A)** *n*, a value proportional to the fraction of cortical neurons connected through the white matter, calculated as (*A*_*W*_/*N*_*G*_) × (*O*_*W*_/*V*_*W*_), is higher in rodents than in primates. **(B)** *a*, a value proportional to the average axonal caliber, calculated as the ratio *V*_*W*_/*O*_*W*_, is higher in primates than in rodents, but increases with number of cortical neurons in rodents to reach primate levels.

Although we cannot determine the real values of *a* and *n*, their scaling can be compared across rodent and primate species, since *V*_*W*_ = *L*.*a* and *A*_*W*_ ~ *n*.*N*.*a*. We find that *n* is about 4× higher in rodents than in primates (Figure 5A), suggesting that a much larger fraction of all cortical neurons are connected through the white matter in rodents than in primates. Moreover, average white matter axonal caliber, *a*, is larger in the sampled primates than in rodents, but while it does not vary significantly across primate species (*p* = 0.9022), it increases with *N*^0.318^_*G*_ in rodents (95% CI, 0.126–0.510, *p* = 0.0451), and approaches primate values in the capybara (Figure 5B).

### Cortical thickness

According to our model of cortical scaling (Mota and Herculano-Houzel, 2012), cortical thickness is a consequence of a combination of factors: number of neurons, average neuronal size in the gray matter (which can be inferred from neuronal density), the fraction of cortical neurons connected through the white matter, and the average caliber of the axons crossing the interface between gray and white matter. Because *V*_*G*_ = *A*_*W*_ × T, and with the relationship between *A*_*W*_ and *N*_*G*_ given above, the relationship T ~ *N*^*t*^_*G*_ can be written as *N*^*t*^_*G*_ ~ *N*^−*d* − *c* − α^_*G*_ (Mota and Herculano-Houzel, 2012). If correct, then the predicted value of the exponent *t* should match the value determined empirically. We find a good match between these values for rodents (predicted *t*, 0.332; observed, 0.427, 95% CI, 0.329–0.525) and primates (predicted *t*, 0.127; observed, 0.109, 95% CI, 0.059–0.159). The good agreement thus supports our hypothesis that cortical thickness is a consequence of the neuronal constitution of the cortical tissue and its relationship with the underlying white matter.

### Cortical gyrification

**Figure 6:**
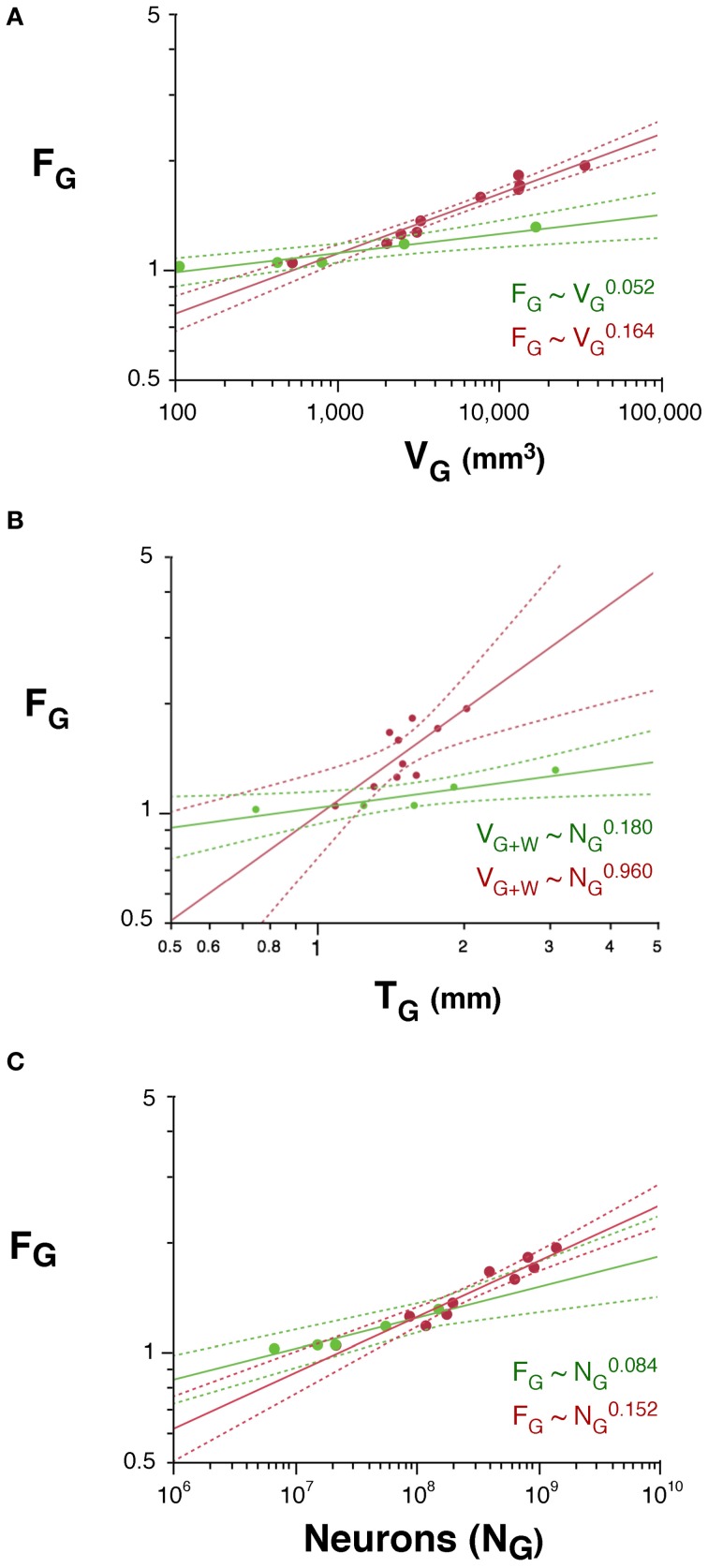
**Scaling of the folding index of the gray matter (F_G_) as a function of gray matter volume (A), number of cortical neurons (B) and cortical thickness (C) in rodents (green) and primates (red).** Each point represents one species. Power functions are plotted for each mammalian order with the respective 95% confidence intervals (dotted lines). Exponents are indicated.

The folding of the cortical surface (F_G_), measured as the ratio between the total pial surface and the surface exposed in gyri, increases slowly across rodent species with *V*^0.052^_*G*_ (95% CI, 0.034–0.070, *p* = 0.0115), and significantly faster across primates, with *V*^0.164^_*G*_ (95% CI, 0.140–0.188, *p* < 0.0001; Figure 6A). As a result, cortices are more folded in primates than in rodents with similar gray matter volumes (Figure 6A) or cortical thickness (T; Figure 6B). In addition, gray matter folding also increases more rapidly with increasing cortical thickness in primates, with T^0.960^ (95% CI, 0.422–1.498, *p* = 0.0073), than in rodents, with T^0.180^ (95% CI, 0.080–0.280, *p* = 0.0376; Figure 6B).

Cortical folding reduces the average length of fibers in the white matter compared to a cortex that had a smooth surface. Given that the average length of axons in the white matter can be written as, $l=\frac{2V_{W}}{A_{W}}\infty N^{\lambda }$ the allometric exponent λ that describes the scaling of *l* with *N* can be determined experimentally from the relationship between the ratio between the measureable values *V*_*W*_/*A*_*W*_ and *N*_*G*_ (Mota and Herculano-Houzel, 2012). In rodents, we find that *V*_*W*_/*A*_*W*_ ~ *N*^2.009^_*G*_/*N*^1.308^_*G*_ ~ *N*^0.701^_*G*_ (95% CI, 0.619–0.783, *p* = 0.0004), in contrast to primates, where *V*_*W*_/*A*_*W*_ ~ *N*^0.207^_*G*_ (95% CI, 0.127–0.287, *p* = 0.0008).

If the volume of the cerebral cortex scaled isometrically, then in rodents we should find *l* ~ *V*^1/3^_*W*_ ~ *N*^2.009/3^ ~ *N*^0.670^, while in primates, λ should vary with *N*^1.197/3^_*G*_ = *N*^0.399^_*G*_. The exponent of 0.207 obtained in primates is significantly smaller than the isometric exponent, which predicts that larger primate cortices have an average axonal length that increases less rapidly than expected for isometricity, and thus is compatible with increasing cortical folding (Herculano-Houzel et al., 2010). In rodents, on the contrary, the calculated λ of 0.701 (95% CI, 0.619–0.783) is not significantly different from the value of 0.670 predicted for isometricity, which indicates that the average axonal length simply increases isometrically, in which case there should be no increase in cortical folding as the rodent cerebral cortex gains neurons. While this is true of the smaller rodent cortices, it is certainly not the case for the two larger species in the sample, the agouti and the capybara, whose cortices are gyrencephalic.

The actual folding of the white matter, F_*W*_, can be determined experimentally for each species as being proportional to *A*_*W*_/*V*^2/3^_*W*_. The exponent of the experimentally obtained scaling of F_*W*_ with N can thus be compared to the predicted exponent given by F_*W*_ ~ N^(1 + *c* + α − 2λ)/3^ in primates, we find an actual F_*W*_ ~ *A*_*W*_/*V*^2/3^_*W*_ ~ *N*^0.513^_*G*_, which matches well the predicted F_*W*_ ~ N^(1 + *c* + α − 2λ)/3^ ~ *N*^0.459^_*G*_. For rodents, we find F_*W*_ to vary with *N*^−0.031^_*G*_ (that is, not to vary significantly with *N*_*G*_), and predict that it varies with *N*^−0.094^_*G*_ (that is, also not significantly with *N*_*G*_). As mentioned above, however, the cortical surface of the two largest rodents in our sample is clearly gyrated, as is the surface of the white-gray matter interface.

We find that the folding of the cortical surface (F_G_), which increases as different functions of *V*_*G*_ in the two orders, increases across rodent species with *N*^0.084^_*G*_ (95% CI, 0.058–0.110, *p* = 0.0082), and significantly faster across primates, with *N*^0.152^_*G*_ (95% CI, 0.122–0.182, *p* < 0.0001; Figure 6C). Because of the very different scaling of *V*_*G*_ with *N*_*G*_ between rodents and primates, the difference between the two orders in the scaling of cortical folding with *V*_*G*_ is even more noticeable, being steeper in primates than in rodents, as noted above (Figure 6A). Thus, cortical folding increases as different functions of the number of cortical neurons across rodents and primates; and, for the same cortical volume, rodents are less gyrencephalic than primates.

Following the same model of cortical gyrification (Mota and Herculano-Houzel, 2012), we predict that F_G_, expressed as the ratio *A*_*G*_/(*V*_*G*_ + *V*_*W*_)^2/3^, scales with N^1 − *d* − *t* − 2*v*/3^. Given the observed values of *d*, *t*, and *v*, we predict that F_G_ should scale in rodents with *N*^0.114^_*G*_, and in primates, with *N*^0.244^_*G*_, which are fairly close to the observed scaling with *N*^0.084 ± 0.013^_*G*_ and *N*^0.152 ± 0.015^_*G*_, respectively.

## Discussion

Cortical expansion in evolution has long been considered a homogeneous process, one supposed to occur through the lateral addition of new columns with a constant number of neurons of which a fixed proportion is connected through the white matter (Prothero, 1997; Zhang and Sejnowski, 2000; Wang et al., 2008). Gyrification supposedly ensues as the cortical surface expands laterally faster than the underlying subcortical and overlying cranial volumes (Welker, 1990)—although a first demonstration that gyrification is actually not a universal function of brain volume was provided by Pillay and Manger (2007), extended recently by Manger et al. (2012). In the same scenario, the volume of the white matter has been considered to scale universally with the volume of the gray matter across mammalian species, with *V*_*W*_ scaling with *V*^1.22^_*G*_ to *V*^1.33^_*G*_ depending on the study (Zhang and Sejnowski, 2000).

In contrast to this traditional view, here we show that the similar scaling of white matter volume with gray matter volume across mammalian orders, as suggested before (Zhang and Sejnowski, 2000), is only apparent: the two distributions are shown here not to be overlapping, with larger volumes of white matter for similar volumes of gray matter in primates than in rodents. More importantly, the apparently similar scaling of white matter volume with gray matter volume masks very different scaling rules of white matter expansion in rodents and primates, with a smaller white matter volume in primates than in rodents for a similar number of cortical neurons; a much faster increase in *V*_*W*_ and the *V*_*W*_/*V*_*G*_ ratio in rodents than in primates as their cortices gain neurons; and a faster increase in cortical folding in primates than in rodents.

In rodents, the addition of neurons to the cerebral cortex is accompanied by decreased neuronal densities in the gray matter (with *N*^−0.640^_*G*_); rapidly increasing cortical thickness (*N*^0.427^_*G*_); unchanging cortical connectivity (the fraction of gray matter neurons connected through the white matter); increasing average axonal caliber in the white matter (*N*^0.318^_*G*_); rapidly increasing white matter volume (*N*^2.009^_*G*_); and slowly increasing cortical folding (*N*^0.084^_*G*_). In primates, in contrast, the addition of neurons to the cerebral cortex is accompanied by unchanging neuronal densities in the gray matter; slowly increasing cortical thickness (with *N*^0.109^_*G*_); decreasing cortical connectivity (*N*^−0.159^_*G*_); unchanging average axonal caliber in the white matter; slowly increasing white matter volume (*N*^1.080^_*G*_); and rapidly increasing cortical folding (*N*^0.152^_*G*_). As a result of these differences, the white matter has a smaller volume in primates than in rodents for a similar number of cortical neurons, and white matter volume increases much more rapidly as neurons are added to the cortex in rodents than in primates, with *N*^2.009^_*G*_ in the former, but only *N*^1.080^_*G*_ in the latter. White matter constitution and expansion is thus much more volume-saving in primates than in rodents.

The strongly hypermetric increasing volume of the white matter with increasing numbers of cortical neurons in rodents, in contrast to the nearly linear scaling in primates, is attributable to the increasing average caliber of axonal fibers in the former on top of the addition of isometrically longer fibers, while in primates the volume of the white matter increases linearly with numbers of cortical neurons due to the addition of longer fibers that become elongated below isometricity and a decreasing connectivity fraction. Decreasing connectivity with growth of the network is a characteristic of small-world networks (Watts and Strogatz, 1998; Argollo de Menezes et al., 2000). While the cerebral cortex has been considered to scale as a small-world network, growing through the addition of nodes that are densely interconnected locally but only sparsely interconnected globally (Changizi, 2001; Karbowski, 2003; Sporns and Kötter, 2004; Sporns and Zwi, 2004), our findings suggest that the rodent cerebral cortex scales differently, with a constant connectivity fraction, as a uniform network would.

Interestingly, the finding that average fiber caliber increases together with increasing *N*_*G*_ in rodents while remaining nearly invariant in primates is qualitatively very similar to what happens with predicted average neuron size in both orders: it increases significantly with *N*_*G*_ in rodents, but does not vary significantly with N across primate species (Herculano-Houzel, 2011). This similarity suggests that there is a link between average neuron size in the gray matter and axon caliber in the white matter.

Additionally, our data implies that a larger fraction of neurons is connected through the white matter in rodents than in primates; and that the average axonal caliber in the subcortical white matter is larger in primates than in rodents, although it increases rapidly in the latter, approaching primate values in the capybara, the largest rodent in our sample. The combination of a relatively much smaller cortical connectivity fraction with only slightly larger average axonal calibers is a possible explanation for the smaller white matter volumes in primate compared to rodent species with similar numbers of cortical neurons (Figure 1D). Thus, primate cortices gain neurons in a more economical fashion than rodent cortices not just in the scaling of white matter volume, but also in the scaling of the absolute white matter volume.

Here we show that cortical thickness scales differently as rodent and primate cortices gain neurons, increasing more rapidly in the former than in the latter. In many models of cortical scaling, cortical thickness is considered to vary inversely with neuronal density (e.g., Prothero, 1997), as required by the hypothesis of a constant number of neurons underneath a unit cortical surface area (Rockel et al., 1980). In contrast, here we show that cortical thickness is not a universal function of neuronal density across mammals, but rather scales more slowly than the inverse of neuronal density in rodents, resulting in a decreasing N/A ratio as numbers of cortical neurons increase, while bearing no relationship to neuronal density in primates (Herculano-Houzel et al., 2008). Thus, cortical thickness is neither a determinant of neuronal density, nor is determined uniquely by it. Instead, our findings fit our recently proposed alternative model (Mota and Herculano-Houzel, 2012): The thickness of the gray matter wall depends not only on average neuronal size in the gray matter but also on their lateral spread, which in turn depends on the fraction of neurons connected through the white matter and the average caliber of the connecting fibers. This can be intuitively understood as the stacking of a number of smaller or larger neurons on top of the gray/white matter interface whose lateral extension depends on the proportion of cortical neurons sending or receiving axons from the white matter, combined to a thinner spreading of cortical neurons over the gray/white matter interface when the average axonal cross-sectional area leaving or entering the white matter is larger. In this scenario, therefore, cortical thickness depends on characteristics of both the gray and white matter.

It has been suggested that a thin cortex of low numbers of neurons correlates best with the higher degree of convolution in some mammalian orders, which is consisten with the notion that these thinner cortices would offer less mechanical resistance to folding, or would impose less pressure on the white matter (Pillay and Manger, 2007). However, not only we show that primate cortices are more folded than rodent cortices over a similar range of cortical thicknesses, but also that folding increases much more steeply with increasing (not decreasing) cortical thickness in primates compared to rodents. These findings contradict the notion that cortical thickness is a direct determinant of cortical folding.

On the contrary, we have previously predicted that cortical thickness is rather a consequence of the parameters that determine cortical folding (Mota and Herculano-Houzel, 2012). According to our model, more highly folded cortices are those that have more neurons connected through the white matter and/or larger average axonal cross-sectional areas, which lead to a thinner cortex for the reasons explained above. We find a connectivity fraction much larger in rodents than in primates for a similar *N*_*G*_, which should lead to a larger folding index in the former for similar numbers of neurons, but this is counterbalanced by larger average axonal calibers in the latter. While these values sometimes average out, such that the capybara cortex is as folded as a primate cortex of similar *N*_*G*_, the very different rates of scaling of connectivity fraction, average fiber caliber, and average fiber length found in rodents and primates predict the comparatively faster increase in cortical folding observed in primates as cortices gain neurons across species and orders.

Importantly, this similarity in the degree of cortical folding between the capybara and some of the smaller primates in our sample, with similar numbers of cortical neurons, might suggest that cortical folding varies as a single, shared function across rodents and primates, and perhaps other mammalian orders. The different scaling rules obtained for rodents and primates in separate suggest, on the contrary, that this is just a coincidence, a crossing point between two unrelated distributions.

Here we show, to the contrary, that gyrification does not vary as a shared, homogeneous function of *N*_*G*_ across rodents and primates. This finding, combined with the order-specific relationships between gray matter volume and its number of neurons (Herculano-Houzel, 2011), explains why folding is not a similar function of *V*_*G*_ across mammalian orders (Pillay and Manger, 2007; Manger et al., 2012). As a consequence, across mammalian orders, and contrary to the traditional expectation, species with more convoluted cortices do not necessarily have more neurons than less convoluted cortices. Indeed, our preliminary data suggest that the highly folded cortices of various artiodactyl species and the elephant have very folded cortices that however hold very few neurons, falling well outside of the relationships found here for rodents and primates (SHH, unpublished observations).

We have previously hypothesized that cortical folding is driven by the folding of the white matter surface, under the tension of its axons (Mota and Herculano-Houzel, 2012). While the folding of the white matter scales across primate cortices with an observed exponent that matches our prediction (*N*^0.513^_*G*_ and *N*^0.459^_*G*_, respectively), in rodent cortices the white matter folding is predicted not to increase with *N*_*G*_. As a consequence, according to our model, the gray matter should thus not become increasingly folded with *N*_*G*_—and yet it clearly does. However, we believe that the apparent failure of the model to predict the folding of large rodent cortices is due to the fact that in our sample, three of the five species (mouse, rat, and guinea pig) are small-brained and practically lissencephalic. Rodent cortices seem to scale without becoming folded up to a certain point, beyond which larger cortices do become increasingly folded. We speculate that, in the smallest rodent cortices, the tension exerted by axons in the white matter might not be enough to pull the gray matter inwards, but in larger rodents that threshold is overcome, leading to folding. The lack of folding in the smallest cortices is actually circumstantial evidence in favor of the push–pull model that we propose, in which the white matter only begins to fold once the traction that its actions exert upon the gray matter exceeds the resistance of the latter to becoming folded inwards. Examination of a larger sample of gyrencephalic rodent cortices should allow us to clarify this issue and determine whether our white matter-based model is also able to predict the folding of the cortical surface within gyrencephalic rodents.

The more volume-saving scaling of the white matter in primates than in rodents that we report has functional consequences: propagation time and computational capacity can be estimated to scale more advantageously in primates than in rodents as the number of cortical neurons increases across species. Propagation time in white matter axons scales with *N*^τ^_*G*_ where τ = λ − α/2 (Mota and Herculano-Houzel, 2012). Given the values of λ and α found for rodents and primates, we obtain a scaling of propagation time with exponents of 0.542 and 0.191, respectively. This suggests that average signal propagation time through the white mater increases far more steeply with *N*_*G*_ in rodent than in primate brains. The comparatively faster scaling of white matter folding in primates, which we propose to result from stronger minimization of axonal lengths under tension, thus bestows upon primate cortices the advantage over rodents of gaining neurons without having white matter signal propagation slowed down as much.

We also predict the computational capacity of the white matter to increase faster in primates than in rodents as their cortex gains neurons. As described before (Mota and Herculano-Houzel, 2012), a rough estimation of computational capacity of white matter connections scales with *N*^θ^_*G*_ with θ = 1 + *c* − τ, which we can estimate to scale in rodents and primates as respectively 0.448 and 0.650. Importantly, however, although computational capacity increases faster in primates than in rodents, in both orders it increases more slowly than the rate at which the cortex gains neurons. Similarly, the computational efficiency of the WM, predicted to scale with N^−2λ − (α/2)^, can be estimated to scale with N^−1.561^ in rodents, and with N^−0.430^ in primates (Mota and Herculano-Houzel, 2012). In both orders, thus, the increase in number of cerebral cortical neurons is accompanied by a decrease in the computational efficiency of the white matter—but this decrease is much faster in rodents.

Finally, our findings of order-specific scaling rules for cortical connectivity through the white matter also have important evolutionary and developmental consequences. Developmental models of evolution must no longer consider that white matter scaling is homogeneous, with similar underlying developmental processes across orders. Our data thus call for a reappraisal of how cortical expansion occurs in development and evolution. We propose that evolutionary expansion of the cortical gray matter, with the accompanying expansion of the subcortical white matter and increased folding of the cortical surface, is governed by similar mechanisms across mammals, albeit with order-specific scaling exponents that lead to order-specific relationships between numbers of cortical neurons and the few parameters that define cortical morphology. According to our model (Mota and Herculano-Houzel, 2012), these are: (1) the number of cortical neurons connected through the white matter, which is the product of the total number of cortical neurons and the connectivity fraction; (2) the average cross-sectional area of axons crossing the white matter; and (3) the shrinkage, under tension, of average axonal length relative to isometry. All these parameters are considered to scale as power functions of *N*_*G*_. Just one further parameter, the scaling of (4) neuronal density with increasing *N*_*G*_, is required to predict both how the thickness of the gray matter varies, and how the folding of the gray matter itself scales. Cortical expansion in each mammalian order would thus scale as a function of *N*_*G*_ with different, order-specific combinations of the exponents *c*, λ and α, leading to cortices that become increasingly folded at different rates as they gain neurons, as long as λ < (*c* + α + 1)/2. Notice that when λ = (*c* + α + 1)/2, folding is predicted to not increase significantly across species with larger cerebral cortices, as was recently suggested to be the case in cetaceans by Manger et al. (2012). Thus, our model also predicts the circumstances in which cortical expansion should occur isometrically, without changes in gyrencephaly. As pointed out by Manger et al. (2012), understanding the mechanisms underlying cortical gyrification requires a multifactorial approach that also takes phylogenetic relationships into consideration. Systematic studies of cortical scaling in large-brained and highly gyrencephalic mammalian orders are underway in our laboratory and should determine whether the different scaling rules that apply to gray and white matter expansion across mammalian orders indeed result, as we predict, from a unified mechanism that involves only a handful of parameters, but which are implemented with different exponents across mammalian orders.

### Conflict of interest statement

The authors declare that the research was conducted in the absence of any commercial or financial relationships that could be construed as a potential conflict of interest.

## Abbreviations

*a*: Average cross-section area of myelinated axons in WM
*A*_*W*_: White-gray matter interface surface area
*A*_*G*_: Total surface area of cerebral gray matter
*F*_*G*_: Cortical folding index
*F*_*W*_: White matter folding index
GM: cerebral cortical gray matter
*l*: Average length of myelinated axons in WM
*L*: Total length of myelinated axons in WM
*n*: GM connectivity through the WM, defined as the fraction of neurons in GM with myelinated axon in WM
*N*_*G*_: Number of neurons in GM
*O*_*W*_: Number of other (non-neuronal) cells in WM
T: Average thickness of cerebral cortex GM
*V*_*G* + *W*_: Volume of cerebral cortex (GM + WM)
*V*_*G*_: Volume of cerebral cortex gray matter (GM)
*V*_*W*_: Volume of cerebral cortex white matter (WM)
WM: subcortical white matter.
